# Ecotoxicological Monitoring of DSS Microorganisms and Quorum Sensing-Mediated Behavior Control over Metal Corrosion of Al, Zn and Fe

**DOI:** 10.3390/toxics14040297

**Published:** 2026-03-29

**Authors:** Raluca Elena Dragomir, Catalina Gabriela Gheorghe, Cristina Maria Dușescu-Vasile, Ana-Maria Manta, Daniela Luminita Movileanu, Dorin Bombos

**Affiliations:** 1 Petroleum Refining Engineering and Environmental Protection Department, Faculty of Petroleum Refining and Petrochemistry, Petroleum-Gas University of Ploiesti, 39 Bucharest Avenue, 100680 Ploiesti, Romania; ralued@yahoo.co.uk (R.E.D.); dmovileanu@upg-ploiesti.ro (D.L.M.); 2 Chemistry Department, Faculty of Petroleum Refining and Petrochemistry, Petroleum-Gas University of Ploiesti, 39 Bucharest Avenue, 100680 Ploiesti, Romania

**Keywords:** biofilm, metal corrosion, *Diatoms*, *Saccharomyces*, *Spirulina*

## Abstract

To evaluate the behavior of industrial equipment from a corrosion point of view, it is mandatory to consider both the material that equipment is made from and the working conditions such as temperature, pH, and the existing microorganisms in the working environment. Our studies regarding ecotoxicological monitoring of biological suspensions *Diatomee*, *Saccharomyces*, and *Spirulina* (DSS) are focused on three directions: (1) the evolution of chemical and biological parameters of the reaction environment (pH, conductivity, TDS, DO, OD), the kinetics of DSS microorganisms’ growing curve; (2) the analysis of biofilm forming on the exposed metallic surface and (3) the analysis of corrosion degree (phenomena) of tested metals in five media, by using the corrosion indices: volumetric index, gravimetric index, and penetration index. The viability of microorganisms in the presence of aluminum, zinc, and iron shows the following sequence: Al*_Diat_* > Fe*_Diat_* > Zn*_Diat_* > Al*_Spir_* > Zn*_Spir_* > Al*_Sach_* > Zn*_Sach_* > Fe*_Spir_* > Fe*_Sach_*. The development of biofilms on the surface of metal plates followed the sequence outlined below: Al*_Diat_* > Fe*_Diat_* > Zn*_Diat_* > Fe*_Spir_* > Zn*_Sach_* > Fe*_Sach_* > Al*_Sach_* > Zn*_Spir_* > Al*_Spir_*. Iron exhibits the most favorable performance, displaying a very low Ip value across all tested environments, including salt water. Aluminum demonstrates sensitivity to specific biological environments, with the highest degree of corrosion observed in *Spirulina*, indicating that not all biological environments confer protection to aluminum. *Diatoms* and *Saccharomyces* suspensions exert an inhibitory effect on corrosion. Zinc is the most susceptible metal, experiencing the greatest corrosion in *Spirulina*, followed by salt water, while biological environments only partially mitigate the corrosion rate.

## 1. Introduction

The aquatic environment contains dissolved ions that have diverse effects on biofauna: temperature increases caused by climate change, specific activities such as industrial waste transport, water pollution due to industry or agricultural activities through excessive use of nutrients or pesticides, fertilizers lead to soil pollution, and water that can weaken the ecological resistance of the environment [1]. Corrosion refers to the total or partial degradation of metals or metal alloys resulting from chemical, electrochemical, or biological interactions with environmental substances. This process can occur in the atmosphere, water, soil, and in technological or household installations. The rate and extent of corrosion are influenced by the chemical composition of the environment and the presence of biological compounds or salts, which may accelerate or inhibit corrosion. Contemporary protective strategies employ inorganic, organic, and environmentally friendly inhibitors to mitigate metal degradation [2,3]. Microorganisms such as bacteria, yeasts, protozoa, and metazoans can induce biochemical oxidation, also known as biocorrosion, by utilizing metal as a culture medium or by degrading surface products. The extent of corrosion in these environments depends on factors including the composition of the reaction medium, pH, oxygen concentration, and the presence of specific microorganisms and algae [4,5,6,7]. The aim of our work was to examine whether biofilms formed on metal surfaces inhibit corrosion in acidic solutions, rather than focusing on microbiological protection against corrosion. The work makes a significant contribution to the development of methods for protecting metals from biocorrosion, and it is also interdisciplinary in nature, combining aspects of ecology, microbiology, and materials science. Microorganisms such as *Diatomee*, *Saccharomyces* and *Spirulina* were selected in this study for their biological and structural properties, in accordance with recommended criteria for assessing biofilm effects on metal corrosion in acidic environments. The three selected microorganisms form stable, adherent biofilms and secrete extracellular polymeric substances (EPSs), including polysaccharides and proteins. These biofilms may reduce the diffusion of aggressive ions (H^+^, Cl^−^) to the metal surface, bind metal ions, and inhibit electrochemical corrosion reactions.

Microorganisms were also selected for their metabolic activity to include additional metabolic protection systems in the evaluation. Their resilience in harsh environments further supports their suitability for experiments in acidic solutions. For corrosion testing we chose DSS suspensions because in the context of environmental biomonitoring, *Diatoms*, yeasts, and cyanobacteria represent complementary model organisms with spread in natural environments. DSS microorganisms have a rapid response to chemical stress, withstand variations in pollutants (heavy metals), are easy to cultivate in the laboratory, and have a short cell growth rate and high cell viability in oxidative stress generated by pollutants in the reaction medium. *Diatoms* and *Saccharomyces* are bioindicators for aquatic ecosystems, react rapidly to changes in the chemical composition of water (pH, organic and inorganic substances), and are part of the “bio” component of anaerobic and aerobic sludges (can withstand low oxygen concentrations). *Spirulina* and *Diatoms* are cyanobacteria found in high salinity environments such as lake benthos, seawater and brackish water. About *Saccharomyces*, it has been reported that there have been approximately 30 yeast species isolated from sediments collected from the ocean floor. It is known that microalgae have reactive groups with active binding sites that can form complexes with pollutants in wastewater [8,9,10,11,12,13,14].

This work investigates the inhibitory effect of biofilms formed on metallic substrates on corrosion processes in acidic media. Diverging from the traditional paradigm of microbiologically influenced corrosion (MIC) as a degradation mechanism, the study examines biofilms as functional interfacial layers that can reduce corrosion rates. The novelty resides in elucidating biofilm-mediated protection mechanisms under low-pH conditions and in advancing bio-inspired corrosion control strategies. Our study redefines microorganisms from a corrosion problem into a corrosion solution, especially in aggressive acidic environments, paving the way for sustainable, bio-based protection technologies.

## 2. General Experimental Design

### 2.1. Analytical Methods

The ecotoxicological monitoring tests were performed to follow three directions: first, we tracked changes in selected chemical and biological parameters in the reaction medium, including pH, conductivity, total dissolved solids, dissolved oxygen, oxygen demand, and the kinetics of DSS microorganism growth [15,16,17,18,19,20,21,22,23,24]. Second, we analyzed biofilm formation on exposed metal surfaces. Third, we assessed metal corrosion by immersion in 3% HCl and analysis of the degree of corrosion of the metals by corrosion indices: volumetric index and penetration index. Corrosion is a chemical process that occurs when metals are exposed to a liquid medium [25,26]. To assess the corrosive or protective properties of various reaction media, 15 bioreactors were monitored over a 28-day period. The period of 28 days was chosen in accordance with the OECD Test Guidelines no. 301 for testing of chemicals, in order to be able to analyze a complete curve of the microbiological development phase of the suspensions analyzed [25].

### 2.2. Preparation of Metal Samples

Parallelepiped metallic specimens, comprising five coupons each of aluminum, zinc, and iron, were machined to precise dimensions (L = 20 mm, W = 10 mm, T = 1 mm) and subjected to initial gravimetric determination. Surface preparation involved alkaline degreasing in a 10% (*w*/*v*) NaOH solution, followed by acid pickling in 0.5 N HCl to remove surface oxides and impurities. The samples were subsequently oven-dried for 1 h and equilibrated to constant mass in a desiccator prior to final weighing.

### 2.3. Materials and Methods

To support experimental determinations, several analytical instruments were employed. Gravimetric measurements were performed using an OHAUS analytical balance, model AX224M (Nänikon, Switzerland). The bioreactors were maintained on an ORBITAL Multi-Shaker (IKA-Verke, Staufen, Germany). Monitoring of microbial viability during the test period was conducted using a CELESTRON digital microscope, model 4434 (Torrance, CA, USA). Microbial growth in the DSS culture was monitored spectrophotometrically using a UV–Vis spectrophotometer (T85+, PG Instruments, Leicestershire, United Kingdom). The measured absorbance values were correlated with cell density (cells·mL^−1^) using McFarland standards. Cell viability was determined through microscopic visualization and quantification in the visual field using a Thoma cell counting chamber (Torrance, CA, USA).

Physicochemical parameters of the culture media such as pH, conductivity, TDS and dissolved oxygen content were recorded using a WTW Inolab Multi 9630 IDS multiparameter (Weilheim Germany). Chemicals were weighed using an OHAUS analytical balance, model AX224M. All the chemicals used in this study were purchased from Sigma Aldrich Chemical Reagents (Sigma-Aldrich, St. Louis, MI, USA).

#### 2.3.1. Preparation of *Diatoms* Suspension

The activated biological sludge was collected from a wastewater treatment plant in the petrochemical sector in Ploieşti, Romania. An aliquot volume of the biological sludge was collected, rinsed several times with tap water (the supernatant liquid was discarded), and centrifuged to achieve a suspended particle concentration of 100 g/L. Aspiration was used to extract the biological suspension containing *Diatoms* cells, which were then submerged in 500 mL of mineral medium (MM): Ca(NO_3_)_2_ × 4H_2_O 4.00 g/L, KH_2_PO_4_ 2.5 g/L, MgSO_4_ × 7H_2_O 5 g/L, NaHCO_3_ 3 g/L, FeNaEDTA 0.45 g/L, Na_2_EDTA 0.45 g/L, H_3_BO_3_ 0.5 g/L, MnCl_2_ × 4H_2_O 0,3 g/L, (NH_4_)_6_Mo_7_O_24_ × 4H_2_O 0.2 g/L, Cyanocobalamin 0.008 g/L, Thiamine-HCl 0.008 g/L, Biotin 0.008 g/L, Na_2_SiO_3_ × 9H_2_O 11.4 g/L. Medium MM was adjusted at pH of 6.5 and autoclaved at 121 °C for 20 min [26,27]. After inoculation of *Diatoms* cells in the sterile culture medium, the biological material was incubated under optimal conditions for adaptation and cell growth for 7 days [28,29,30,31].

#### 2.3.2. Preparation of Yeast Suspension *Saccharomyces* sp.

The yeast cell culture was obtained from the Culture Collection of Petroleum-Gas University of Ploiesti. It is a representative of a category of eukaryotic unicellular microorganisms that are spherical in shape. Yeast cell cultures were grown in specific medium (YM) prepared from 10 g/L yeast extract, 20 g/L peptone, 20 g/L dextrose, 20 g/L NaH_2_PO_4_. The substances were dissolved in distilled water; the pH was adjusted to 6.5 and YM was autoclaved at 121 °C for 20 min [26,27]. After inoculation of *Saccharomyces* sp. cells in the sterile culture medium, the biological material was incubated under optimal conditions for adaptation and cell growth for 7 days [28,29,30,31].

#### 2.3.3. Preparation of Algal Suspension *Spirulina* sp.

The algal cell culture was obtained from the Culture Collection of Petroleum-Gas University of Ploiesti. Algal suspension was cultivated in Erlenmeyer flasks containing Zarrouk medium (ZM) (NaHCO_3_18 g/L, NaNO_3_ 2.5 g/L, K_2_HPO_4_ 0.5 g/L, K_2_SO_4_ 1.0 g/L, FeSO_4_ × 7H_2_O 0.01 g/L, Na_2_EDTA·2H_2_O 0.08 g/L, NaCl 1.0 g/L, CaCl_2_ × 2H_2_O 0.04 g/L, MgSO_4_ × 7H_2_O 0.2 g/L) and 1 mL micronutrient solution (H_3_BO_3_ 2.8 g/L; MnCl_2_× 4H_2_O 1.8 g/L, CuSO_4_ × 5H_2_O 0.08 g/L; ZnSO_4_ × 7H_2_O 0.2 g/L; Na_2_MoO_4_·2H_2_O 0.4 g/L). For Zaruk, medium pH was adjusted to 9.5, and it was sterilized by autoclaving for 15 min at 121 °C [26,27]. After inoculation of *Spirulina* sp. cells in the sterile culture medium, the biological material was incubated under optimal conditions for adaptation and cell growth for 7 days [28,29,30,31].

### 2.4. The Research Method

The first stage of the research was the cellular development phase of DSS. The objective at this stage was to obtain young biomass in the exponential growth phase. During the DSS cell development stage, Erlenmeyer flasks (bioreactors) containing media specific to the microorganism were used according to Section 2.3.1, Section 2.3.2 and Section 2.3.3 under laboratory conditions in specific media for each microorganism. Biological suspensions were agitated and incubated under white light for 7 days, with a 12 h photoperiod and light intensity ranging from 60 to 120 µE∙m^−2^∙s^−1^. The bioreactors were maintained on an ORBITAL Multi-Shaker at 50 rpm to ensure optimal aeration at 28 ± 1 °C [28,29,30,31]. Cell viability was assessed throughout the experiment using a Celestron Microscope (4434, Torrance, CA, USA).

**Figure 1:**
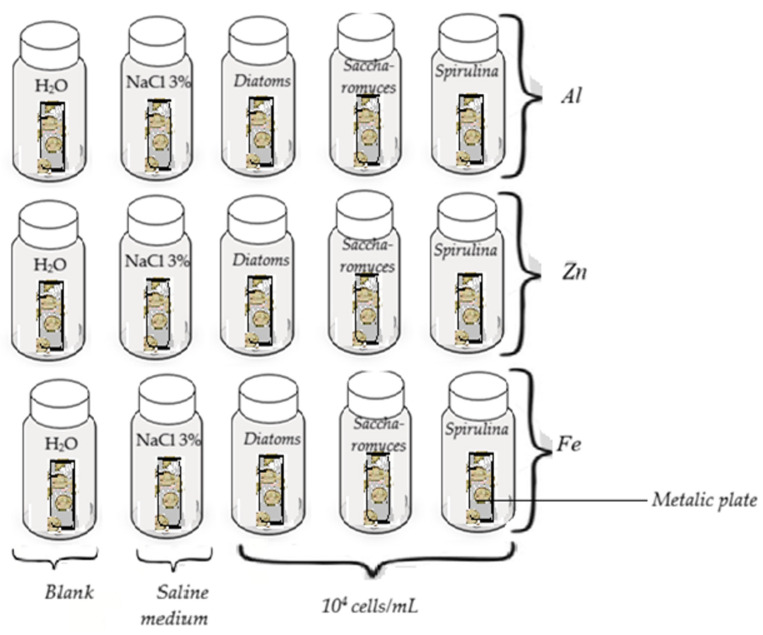
The biotic and abiotic components of the analyzed bioreactors.

The second stage of the experiment was planned as follows: 15 Erlenmeyer flasks (bioreactors) were used, shown schematically in Figure 1.

The bioreactors were marked as follows: Series A, B, and C. Each series contains 5 Erlenmeyer flasks (bioreactors) coded: Series A: Al*_H_*_2_*_O_*, Al*_ss_*, Al*_Diat_*, Al*_Sach_*, Al*_Spir_*; Series B: Zn*_H_*_2_*_O_*, Zn*_ss_*, Zn*_Diat_*, Zn*_Sach_*, Zn*_Spir_*; Series C: Fe*_H_*_2_*_O_*, Fe*_ss_*, Fe*_Diat_*, Fe*_Sach_*, Fe*_Spir_* (Table 1). In the bioreactors of series A, Al metal plates were introduced, in series B, Zn plates were used; in series C, Fe plates were introduced. Each series contained a blank reference bioreactor that contained 15 mL distilled water (marked according to the series from which it comes: Al*_H_*_2_*_O_*, Zn*_H_*_2_*_O_*, Fe*_H_*_2_*_O_*,) and one bioreactor that contained 15 mL 3% NaCl saline solution (Al*_ss_*_,_ Zn*_ss_*_,_ Fe*_ss_*). In the bioreactors coded Al*_Diat_*, Zn*_Diat_*, Fe*_Diat_*, respectively, Al*_Sach_*, Zn*_Sach_*, Fe*_Sach_*, and Al*_Spir_*, Zn*_Spir_*, Fe*_Spir_*, 15 mL of inoculum suspension for each bioreactor with one type of microorganism, *Diatoms*, *Spirulina* and *Saccharomyces*, containing 10^4^ cells/mL in the exponential growth phase was added [25,26,27]. The bioreactors were closed with screw caps. The testing was carried out over 28 days under identical conditions across for all bioreactors which were mechanically agitated using an orbital shaker operated at 50 rpm and maintained at 30 °C [28,29,30,31]. These conditions were selected to enhance mass transfer, promote gas exchange, and ensure consistent interaction between the reaction medium and the exposed metallic surfaces.

**Figure 2:**
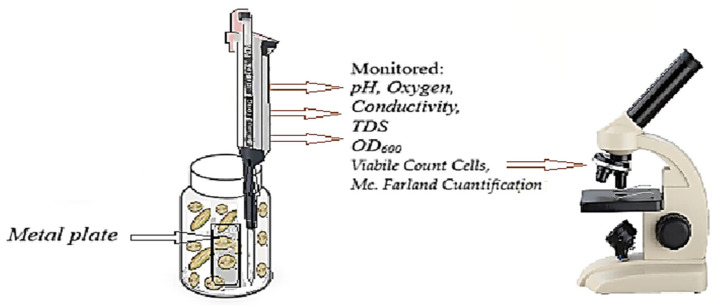
Presentation of experimental activities for each bioreactor during 28 days of exposure to specific environment.

**Figure 3:**
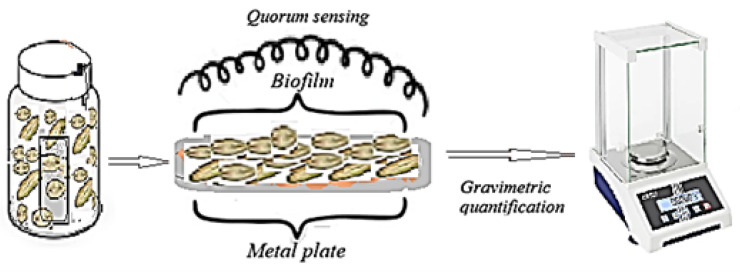
Presentation of the quantification stage of the biofilm formed on the surface of each metal plate in the analyzed bioreactors.

The experimental investigation was structured along three principal directions: (i) monitoring the temporal evolution of selected physicochemical and biological parameters of the reaction medium (Figure 2); (ii) evaluating the kinetics and extent of biofilm development on the exposed metallic substrates (Figure 3); and (iii) assessing the corrosion degree of the tested metals in the presence of the five distinct media. Accordingly, the reaction medium was periodically analyzed for pH, electrical conductivity, total dissolved solids (TDS), and dissolved oxygen (DO) concentration. Microbial growth dynamics were monitored spectrophotometrically (by measuring optical density at 600 nm (OD_600_)), enabling estimation of biomass concentration and cell density (cells mL^−1^).

Throughout the 28-day experimental period, the cell viability was evaluated through microscopic visualization and quantification within the field of view using a Thoma cell counting chamber. Cellular structures were examined using optical microscopy and scanning electron microscopy (SEM).

After 28 days of exposure, the metal coupons were retrieved from the bioreactors, gently dried at 25 °C, and subjected to gravimetric analysis to quantify the mass of biofilm (biological mass deposited after 28 days of exposure and potential corrosion products) accumulated on the surface (Figure 3). The difference between the final and initial constant mass was used to determine biofilm biomass accumulation, in accordance with established methodologies [25,26,27].

At the end of the exposure period in the biological medium, after the gravimetric measurement of the deposited biofilm, each metal plate was analyzed for corrosion resistance in acid medium [32,33,34,35,36].

**Figure 4:**
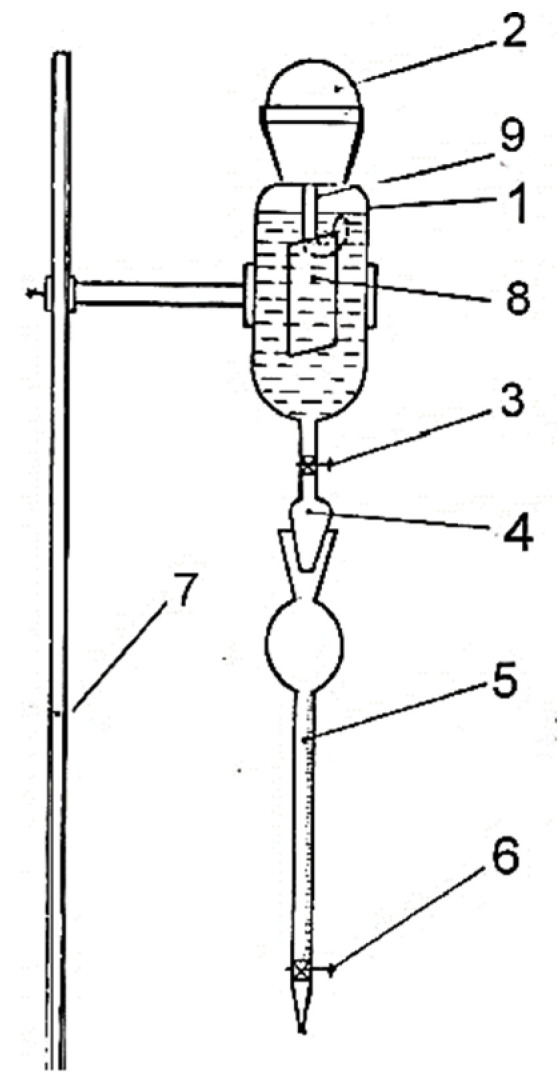
Laboratory installation for determining metal corrosion 1—reaction vessel; 2—ground plug for tightness of the installation; 3—tap; 4—ground; 5—burette; 6—tap; 7—metal stand; 8—metal specimen; 9-metal specimen clamping system.

Corrosion was assessed as a chemical performance parameter, allowing evaluation of whether the reaction medium in contact with the metal substrates facilitated the development of a biofilm exhibiting either protective (passivating) or corrosive behavior. Following this stage, the corrosion behavior of the metallic specimens was evaluated by exposing each plate to a 3% HCl solution, as illustrated in Figure 4. The metal sample was immersed, by hanging in 3% HCl solution placed in a cylindrical separatory funnel with a ground stopper at the top and a tap at the bottom. The funnel was connected to a graduated burette (cm^3^) equipped, in turn, with a stopcock. The metal sample was measured before immersion to determine the surface area that comes into contact with the 3% HCl solution. The cylindrical funnel is filled with the 3% HCl solution in a proportion of ¾ of the capacity of the vessel. The cylindrical funnel is sealed after the complete immersion of the metal sample. The stopcock of the cylindrical funnel is opened and the level of the solution in the burette is read, which is the level at time 0. The stopcock of the burette is closed. After the start of the experiment, the level of the liquid in the burette is read every 10 min, for 1 h.

The experiments were conducted under controlled laboratory conditions (T = 18 °C; P = 741 mm Hg). Corrosion assessment was based on volumetric quantification of hydrogen gas evolved during the metal–acid reaction. The volume of displaced solution, corresponding to the hydrogen generated, was measured and used as an indirect indicator of the extent of metal dissolution. This approach relies on the stoichiometric relationship between metallic oxidation and hydrogen evolution in acidic media, thereby enabling comparative evaluation of corrosion intensity among the tested samples (Equations (1)–(3)).

(1) $$2\mathrm{Al}+6\mathrm{HCl}\to 2{\mathrm{AlCl}}_{3}+3H_{2}↑$$

(2) $$\mathrm{Zn}+2\mathrm{HCl}\to {\mathrm{ZnCl}}_{2}+H_{2}↑$$

(3) $$\mathrm{Fe}+2\mathrm{HCl}\to {\mathrm{FeCl}}_{2}+H_{2}↑$$

#### 2.4.1. Monitoring of Ph, Oxygen and Electrical Conductivity Evolution

The pH and electrical conductivity were continuously monitored in the bioreactors to evaluate physicochemical changes in the biological suspension following the introduction of the metallic plates [34,35,36,37,38,39]. Measurements were performed daily over the 28-day experimental period using electrochemical sensors (WTW, Inolab MULTI 9630, Weilheim Germany), ensuring consistent and reproducible monitoring of variations in acid–base balance and ionic strength within the system.

#### 2.4.2. Total Dissolved Solids (TDS) Assessment

In the present study, TDS was employed to quantify the total load of metal ions released from the metallic plates, in addition to all other dissolved chemical species generated during the reactions within the medium [40].

TDS was estimated using the conventional conductivity-based conversion approach, applying a factor of 0.67 according to the following equation (Equation (4)):

(4) $$TDS\;\left(mg/L\right)=Conductivity\;\left(\mathrm{mS}/\mathrm{cm}\right)\times 0.67$$

#### 2.4.3. Optical Density (OD_600_) and Viable Counts Measurements

Microbial growth in the DSS culture was monitored spectrophotometrically by measuring the optical density at 600 nm (OD_600_). Daily, over the 28-day experimental period, 1 mL aliquots of the cell suspension were withdrawn from each bioreactor and analyzed using a UV–Vis spectrophotometer (T85+, PG Instruments, Leicestershire, United Kingdom). The measured absorbance values were correlated with cell density (cells·mL^−1^) using McFarland standards. McFarland standards were prepared in ultrapure water as follows: 1% sulfuric acid (Sigma Aldrich, St. Louis, MO, USA) and 1% barium chloride (Sigma Aldrich, St. Louis, MO, USA). Barium sulfate precipitate was measured spectrophotometrically at 600 nm. 

#### 2.4.4. Corrosion Volumetric Tests

The volumetric method was used to determine the corrosive behavior of metals in 3% HCl solution. The method consists in determining the corrosion rate by monitoring, over time, the volume of hydrogen produced by the corrosion reaction, with the help of which the mass of material lost, respectively the corrosion rate, is calculated, in mm/year. The volumetric method for determining the corrosion rate is a specific one, and can be applied only if the corrosion reaction leads to the release of hydrogen as a by-product of this process. This method is used in particular to verify the efficiency of corrosion inhibitors or to establish the effectiveness of some protective anti-corrosion coatings [41,42,43,44,45].

#### 2.4.5. Determination of Volumetric Index

Based on the volumes of hydrogen released during the experiment, the volumetric index, *Kv*, is calculated according to Equation (5).

(5) $$K_{V}=\frac{v}{S\times t}\;,m^{3}/\left(m^{2}\;\times \;h\right)$$

where v is the volume of released gas due the reaction between metal and 3% HCL solution; S is the metal sample surface in contact with HCl solution, and t is the time of contact between metal sample and acid solution.

The volume of gas released during the corrosion reaction is converted into volume under normal temperature and pressure conditions according to Equation (6):

(6) $$V_{0}=\frac{\left(P-P_{H_{2}O}\right)\times T_{0}}{P_{0}\times T}$$

where P is the atmospheric pressure, read at the barometer, mmHg (torr); $P_{H_{2}O}$ is the partial pressure of water vapor at the temperature at which the experiment takes place, mmHg; $P_{0}$ is the normal pressure, 760 mmHg; *T* is the temperature at which the experimental determinations take place (273 + t °C); $T_{0}$ is the normal temperature, 273 K. Based on the volumetric index, the mass loss of the metal sample is calculated over time.

#### 2.4.6. Determination of Gravimetric Index and the Mass of Metal Consumed in the Corrosion Reaction

Based on the volumetric index, the mass loss of the metal sample over time is calculated. The gravimetric index is calculated based on the reaction presented in Equation (7):

(7) $$\mathrm{Me}+\mathrm{yHCl}\to \mathrm{MeCy}+y/2H_{2}↑$$

The argument regarding the chemical reaction that occurs depending on the metal analyzed (Equation (8)) and the calculation method for the mass of metal consumed in the corrosion reaction is as follows:

A g Me...................................*y*/2 × 22.4 L H_2_

a g Me.......................................................V_0_

(8) a=V0×A/y2×22.4, g

where *V*_0_ is the volume of hydrogen released under normal conditions, calculated with Equation (6), and *A* is atomic mass of the metal.

The gravimetric index is calculated according to Equation (9).

(9) $$K_{g}=\pm a/\left(S\times t\right)\;,\;g/\left(m^{2}\;\times \;h\right)$$

where a represents the mass of metal consumed in the corrosion reaction, S denotes the surface area of the specimen (m^2^), and t corresponds to the exposure time of the metal in the solution (h).

#### 2.4.7. The Penetration Index

For uniform corrosion affecting the entire metal surface, the corrosion rate was quantified using the penetration index ($I_{P}$), which represents the average reduction in metal thickness over a given exposure period, as defined in Equation (10). The penetration index provides a quantitative measure of material degradation, enabling assessment of durability and prediction of service life under the specified experimental conditions.

(10) $$I_{P}=\frac{K_{g}}{\rho }\times 8.76,\;\mathrm{mm}/\mathrm{year}$$

where $K_{g}$ represents the gravimetric index (g/m^2^ × h), ρ denotes metal density (g/cm^3^) and 8.76 is a constant derived from the total number of hours in a year (8760 h).

### 2.5. Statistical Data Process

All measurements were performed in four replicates (*n* = 4). Data are expressed as mean ± SD. Differences between treatments (Zn, Fe and Al) and the control group were analyzed using one-way ANOVA test from Microsoft 365 Excel^®^ v.2023 (Microsoft Corporation, Redmond, WA, USA). Statistical significance was considered at *p* < 0.05 [28,29,30,31].

## 3. Results and Discussion

Bacteria and other microorganisms can cause microbiological corrosion through the interaction of organisms responsible for the corrosion of metallic materials. Microorganisms that can cause such chemical reactions are sulfate-reducing bacteria, sulfur-oxidizing iron-reducing bacteria, carbon dioxide-fixing algae, etc. For instance, *Diatoms* algae can transform carbon dioxide dissolved in water into oxygen, which acts as an oxygen source, and supports nutrient cycling in marine waters. Metal dissolution processes may lead to the release of metal ions into the medium, which can exert either inhibitory effects on microbial growth or, conversely, act as micronutrients that stimulate biomass development, depending on their concentration and speciation [40,41,42,43].

The reaction medium, pH, presence or absence of oxygen and the presence of bacteria, algae, protozoa, and metazoans all influence the corrosion of metals exposed to these environments [43,44,45,46,47,48]. A biocorrosion reaction is the oxidation–reduction reaction in which Fe^2+^ is oxidized to Fe^3+^ (Equations (11) and (12)) in the presence of dissolved oxygen (by aeration of the liquid medium), followed by precipitation [45].

(11) $${\mathrm{Fe}}^{2+}+\frac{1}{4}O_{2}+H^{+}\to {\mathrm{Fe}}^{3+}+\frac{1}{2}H_{2}O$$

(12) $${\mathrm{Fe}}^{3+}+3\;{\mathrm{OH}}^{-}\to \mathrm{Fe}{\left(\mathrm{OH}\right)}_{3}↓$$

Corrosion inhibitors adsorb at the metal/solution interface, altering the metal’s structure and zeta potential. For example, polar ions or molecules interact with metal surfaces, changing the metal/solution interface (Equation (13)):

(13) $$M{\left(nH_{2}O\right)}_{adsorbed}+Inhibitor_{\left(sol\right)}\to MI_{ads}+nH_{2}O_{\left(sol\right)}$$

The adsorption of an inhibitor alters the solvent’s dielectric properties. The hydrophobic groups of corrosion inhibitors interact with the metal surface and become adsorbed. Furthermore, the hydrophobic groups of free inhibitor molecules in solution associate with those already adsorbed to the metal, forming semimicelles or passive films. In neutral aqueous solutions, corrosion inhibition is accomplished using oxygen-consuming agents, such as SO_3_^2−^ solutions, which react with O_2_ to produce SO_4_^2−^ (Equation (14)):

(14) $$2{\mathrm{SO}}_{3}^{2-}+O_{2}\to 2{\mathrm{SO}}_{4}^{2-}$$

The presence of heavy metals in the aquatic environment disrupts the physiological balance of the microbiota by accumulating them in living tissues. For example, Cu(II) disrupts the function of microorganisms’ cell membranes due to its redox potential. The corrosion of steel pipes protected with metal coatings immersed in various aquatic environments is also influenced by the presence of chlorides and sulfates, as well as by the environment’s pH. Metals exposed in terrestrial water can be affected by the composition of the liquid medium, which may contain dissolved salts, colloidal substances, and calcium, magnesium, and manganese salts. Seawater, in addition to dissolved salts, also contains NaCl at concentrations ranging from 0.3% to 15%, a chemical composition that can influence the corrosion of metals exposed to these environments [49,50].

Quorum sensing (QS) is a communication mechanism between microorganisms that responds to the chemical composition of the exposure environment and to the time and conditions of exposure, and is dependent on the density of the microbiological population. Under certain conditions, microorganisms can form biofilms, produce pigments, and form spores or agglomerates (flocs) that protect cells from the attack of toxic substances in the environment [51,52,53]. Biofilms develop at a range of environmental interfaces, such as solid/gas, liquid/liquid and solid/liquid interactions. Hydrogen bonds, ionic interactions, and van der Waals forces maintain their structure and promote surface adhesion. Biofilms consist of cellular biomass, water, nutrients, dissimilation products, and reaction products formed in association with abiotic components, such as metal surfaces. The chemical composition of the surrounding medium influences biofilm formation on metal plates.

### 3.1. Physicochemical Parameters: The Evolution of pH, Dissolved Oxygen (DO) Content, Conductivity, Total Dissolved Solids (TDS), Measured in Biological Cultures of DSS in the Presence of Al, Zn, and Fe

The pH evolution in the 15 biological environments was monitored using WTW sensors, with daily measurements over 28 days [25,30,43]. All bioreactors were carefully monitored because the pH value, together with the oxygen value, is a very important indicator to ensure the biological suspension is an environment conducive to cell development. In general, during the 28-day experiment, all suspensions maintained a pH within the neutral range, with slight tendencies toward increased acidity. Oxygen had low values with a decreasing trend. We assume that this was due to chemical oxidation reactions on the metal ions, generated by chemical species in the biological environment, by cellular metabolic reactions, or by corrosion processes.

**Figure 5:**
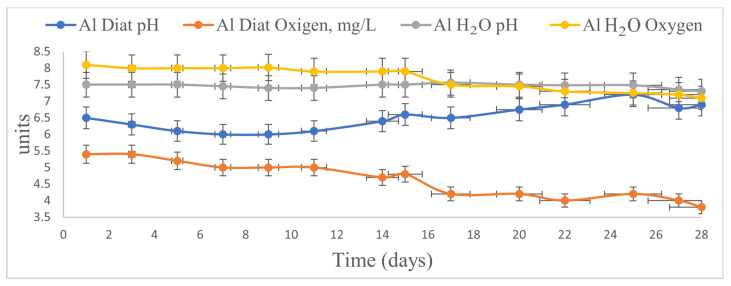
Evolution of pH and oxygen in the bioreactor with *Diatoms* suspension in the presence of the Al plate (measured values are represented with standard deviation, *n* = 4 and trendline).

The *Diatom* is considered an alga that can thrive in environments with low oxygen levels. During the experiment, in combination with the Al plate, the parameter showed a decreasing average; the values were in the range of 5.5–4. Dissolved oxygen values in the blank systems showed a decreasing trend, with ranges of 8.1–7.1 mg/L (Al*_H_*_2_*_O_*). Regarding pH, values ranged from 6.5 to 5.9 until day 10, after which they increased slightly to 7 at the end of the experiment (day 25) (Figure 5). In the blank bioreactor, the pH values varied slightly over the 28-day period: 7.5–7.3 for Al*_H_*_2_*_O_*. The Zn plate maintained the pH of the *Diatoms* algal medium at 5.5–5.8 In the blank bioreactor, the pH values varied slightly over the 28-day period: 7.7–8.3 for Zn*_H_*_2_*_O_* and oxygen 5.5–5.0 for Zn*_H_*_2_*_O_*.

Regarding the Fe plate, during the experiment in the algal environment of *Diatoms*, the pH was maintained at 6–6.5 compared to the variation in pH in the blank bioreactor of 6.4–5.5 mg/L (Fe*_H_*_2_*_O_*), and the oxygen at 5.0–2.8 for the Fe*_Diat_* compared to the variation in the blank bioreactor (oxygen 5.0–3.8 for Fe*_H_*_2_*_O_*). Regarding *Spirulina* algae, in the presence of the Al plate, the pH ranged from 6.5 to 8.5, and the oxygen concentration ranged from 3 to 5 mg/L compared to the variation in the blank bioreactor: oxygen 8.1–7.1 mg/L (Al*_H_*_2_*_O_*) and pH 7.5–7.3 for Al*_H_*_2_*_O_*. The Zn plate maintained the pH of the environment with *Spirulina* between 5.5 and 6.2, and the oxygen value was maintained between 5.8 and 5.5 compared to pH 7.7–8.3 for Zn*_H_*_2_*_O_* and oxygen 5.5–5.0 for Zn*_H_*_2_*_O_*. In the presence of Fe, in *Spirulina* algae medium, the pH was in the range 5.8–6.5, and the oxygen was between 5.6 and 4.8 compared to the blank (6.4–5.5 mg/L-Ph (Fe*_H_*_2_*_O_*) and 5.0–3.8 oxygen for Fe*_H_*_2_*_O_*).

The biological environment of the *Saccharomyces* suspension showed pH values ranging from 6.5 to 5.5 in the presence of the three metal plates (Al, Zn, and Fe). Dissolved oxygen values ranged from 5.6 to 5.2 mg/L in the presence of Al, 5.6 to 4.4 mg/L with Zn, and 5.5 to 5.0 mg/L with Fe. A slight increase in pH was observed after 16 days of the experiment, followed by a decrease after 21 days.

In the bioreactors containing saline solution, pH values ranged between 7.2 and 7.8 (Al*_SS_*), 8.5 and 8.3 (Zn*_SS_*), and 7.7 and 7.6 (Fe*_SS_*). Dissolved oxygen values were 7.8–7.3 mg/L (Al*_SS_*), 4.9–3.9 mg/L (Zn*_SS_*), and 8.2–6.1 mg/L (Fe*_SS_*).

Overall, the presence of metal plates influenced the physicochemical parameters of the medium, with more pronounced variations observed in the biological systems compared to the blanks.

**Figure 6:**
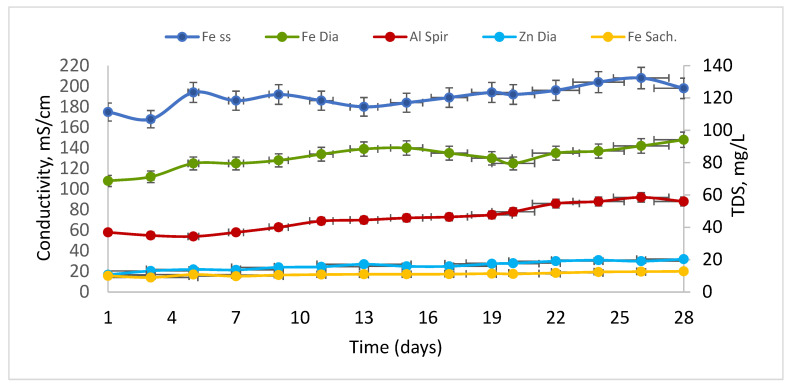
Conductivity measurements corresponding to total dissolved solids (TDS) (measured values are presented with standard deviation (*n* = 4) and trendline).

The highest electrical conductivity was recorded in the Fe-containing saline solution suspension, with values ranging from 160 to 200 mS/cm, and a trendline 170–190 mS/cm corresponding to the total dissolved solids (TDS) concentrations trendline of 115 to 135 mg/L. The *Spirulina* suspension exposed to Al showed intermediate conductivity values of 50–70 mS/cm and TDS levels between 35 and 50 mg/L. Overall, conductivity values remained relatively stable during the initial phase of the experiment. Zn*_Dia_*, Fe*_Sach_*, Al*_Spir_* decreased progressively until day 14 (Fe*_ss_*), and then showed a slight increase toward the end of the experimental period (day 26, Fe*_ss_*) (Figure 6). In contrast, the *Saccharomyces* suspension containing Fe ions and the *Diatoms* exposed to Zn exhibited the lowest conductivity values, ranging from 10 to 12 mS/cm (Fe*_Sach_*) and 20 to 30 mS/cm, respectively (Zn*_Dia_*). Blank bioreactors consistently showed the lowest conductivity values among all experimental systems, with measurements of 34–43 µS/cm (Al*_H_*_2_*_O_*), 24–39 µS/cm (Zn*_H_*_2_*_O_*), and 99–145 µS/cm (Fe*_H_*_2_*_O_*).

### 3.2. Biological Parameters: Optical Density (OD_600_), Viable Count Cells and McFarland Quantification

**Figure 7:**
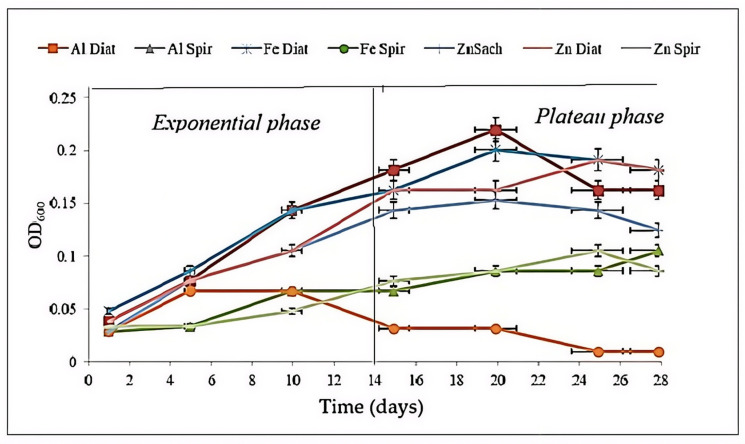
Change in optical density throughout the testing period (measured values are presented with standard deviation (*n* = 4)).

**Figure 8:**
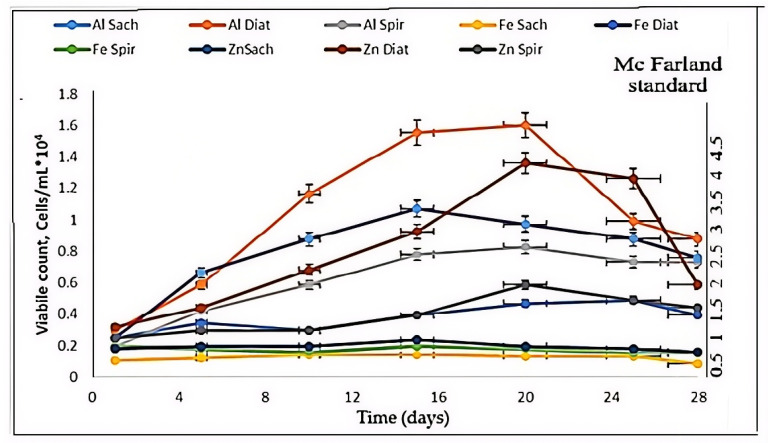
Spectroscopic measurements of cell density (measured values are presented with standard deviation (*n* = 4)).

Cell density was monitored throughout the experimental period to evaluate the development of microorganisms within the bioreactors (Figure 7). Aliquots were periodically collected from the biological suspensions and quantified microscopically using a Thoma counting chamber. The temporal evolution of microbial growth is presented in Figure 8. After approximately half of the experimental period, the *Diatoms* suspension in the presence of Zn and Fe plates exhibited the most pronounced cellular development, reaching a maximum optical density (OD) of 0.23 on day 20. Similarly, the *Diatoms* suspension exposed to Fe showed continuous growth until day 22, attaining an OD of 0.22. In the presence of Al, the *Diatoms* culture also demonstrated progressive growth, reaching an OD of 0.20 by the end of the experiment. Regarding cell viability, the *Diatoms* suspension was the most metabolically active among all tested cultures. The highest viability was recorded between days 16 and 20, with viable cell counts ranging from 1.0 to 1.8 × 10^4^ cells/mL (corresponding to 3–4.5 McFarland units) [54]. By the end of the experiment, cell concentration slightly decreased to 1.0–1.3 × 10^4^ cells/mL (2–3 McFarland units). The lowest cell viability was observed in the *Saccharomyces* suspension exposed to Fe, followed by the *Spirulina* suspension in the presence of Fe, indicating a stronger inhibitory effect of iron under the tested conditions.

*Saccharomyces* yeast cultures exhibited minimal cell growth in the presence of the Fe plate, with an initial cell viability of 0.15 × 10^4^ cells/mL and only slight variation observed throughout the experimental period. This corresponded to an approximate turbidity of 0.5 McFarland, theoretically equivalent to a standard concentration of 1.5 × 10^8^ cells/mL (presented in Figure 8).

In contrast, *Spirulina* demonstrated enhanced cell growth in the presence of the Al plate, reaching approximately 2.5 McFarland units, with an initial cell viability of 0.1 × 10^4^ cells/mL.

Comparative analysis of the growth curves indicates that the *Diatom* suspension exhibited the most significant cell development and viability in the presence of Al and Zn plates, followed by Fe. Conversely, *Spirulina* showed the weakest cellular development in the presence of the Fe plate, suggesting a stronger inhibitory effect of iron on this microorganism under the tested conditions [28,29,30,31].

### 3.3. Biofilm Evolution: Quorum Sensing (QS) Control of Bioreactors Behaviors

DSS form biofilms on metal surfaces, which generate a series of redox reactions at the cellular level. The toxicity of metal ions generates a possible defense mechanism of microorganisms that through local electrochemical phenomena secrete extracellular polymers that could become the support for the development of bacteria that intervene in corrosion. Microbiological synergism is observed by the formation of the adherent film more accentuated in the *Diatom* culture perhaps because they are photosynthetic algae with cell cavities-frustules that help the biofilm adhere to the metal. Quorum sensing (QS) represents a mechanism of chemical communication dependent on cell density, through which microorganisms coordinate gene expression at the biofilm level. The biofilm formed on metal plates is likely to be responsible for the generation of autoinducers released by Gram + or Gram— bacteria that diffuse into the extracellular environment and activate specific receptors for metal ions, thus leading to microbiological corrosion.

**Figure 9:**
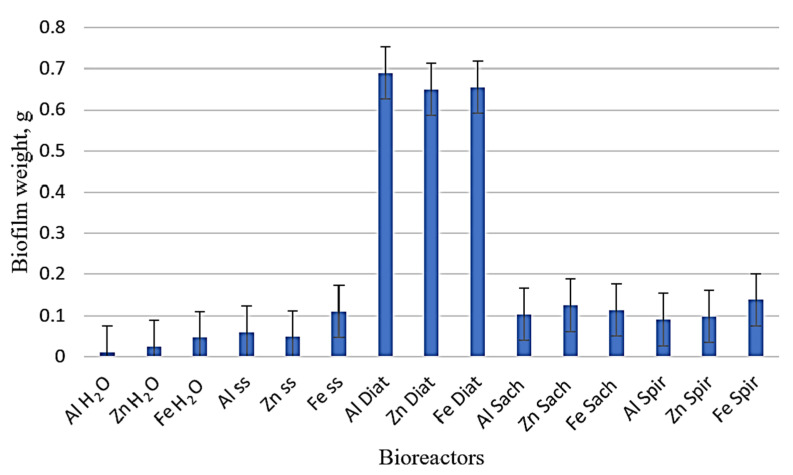
Biofilm mass generated via quorum sensing in the presence of Al, Zn, and Fe compared to the variation in the blank bioreactor after 28 days of experiment (measured values are presented with standard deviation (*n* = 4)).

Biofilm formation [54,55,56,57,58,59,60,61] results from the adhesion of microbial cells to the metal surface and is influenced by several physicochemical and biological factors, including pH, ion concentration in the reaction medium (as reflected by conductivity), gas exchange (CO_2_ and O_2_), metal type, surface roughness, and contact angle. As cell concentration increases, adhesion to the developing biofilm matrix is enhanced through quorum sensing (QS) mechanisms, which regulate collective microbial behavior and extracellular polymeric substance (EPS) production. Figure 9 illustrates the variation in biofilm mass formed in the different reaction media over the course of the experiment.

*Diatoms* algae were able to form stable and strongly adherent biofilms on the surfaces of all three studied metals (Al, Zn, and Fe). As shown in Figure 9, the biofilm mass formed by the *Diatoms* cultures on the metal plates was approximately 6–7 times greater than that produced by *Saccharomyces* or *Spirulina* under similar conditions [13,28,29,30]. In the blank systems (distilled water and saline solution), the observed deposits were primarily the result of physicochemical reactions between the metal surface and the aqueous medium. However, in the presence of microorganisms, biofilm formation was predominantly biologically mediated, although it may have also incorporated corrosion products and released metal ions.

The formation of biofilms can have a dual effect on metal surfaces. On one hand, the biofilm layer may increase resistance to corrosion by acting as a physical barrier. On the other hand, it may accelerate metal degradation by consuming reaction products or metal ions and by promoting localized corrosion processes [18,19,20,21,22]. A possible mechanism for metal degradation involves the enzymatic activity of the biofilm, which can transform the substrate into secondary metabolites that further influence surface chemistry. The *Diatoms* biofilm, due to its photosynthetic metabolism, acts as a localized oxygen generator. This oxygen production may initiate or enhance additional electrochemical reactions at the metal surface. The significant increase in *Diatoms* biofilm mass is consistent with the higher optical density (OD) values recorded for this culture, reflecting enhanced growth and biomass accumulation. Moreover, the *Diatom* culture exhibited a higher degree of flocculation compared to the other tested microorganisms, contributing to greater surface attachment and biofilm development. Consequently, the *Diatoms* suspension demonstrated stronger quorum sensing (QS) activity, associated with increased biomass and extracellular polymeric substance (EPS) production, leading to thicker and more cohesive biofilms [14,17]. The study demonstrates that microbiologically influenced corrosion (MIC) is a selective phenomenon, dependent on the type of metal, with the impact of the biological environment governed by the specific interaction between microorganisms and the metallic substrate rather than by a universal biological effect. The evolution of biofilm mass on the metal plates followed the order: Al*_Diat_* > Fe*_Diat_* > Zn*_Diat_* > Fe*_Spir_* > Zn*_Sach_* > Fe*_Sach_* > Al*_Sach_* > Zn*_Spir_* > Al*_Spir_*. The development of biofilms in the aqueous environments of the bioreactors was influenced by reaction parameters such as pH, dissolved oxygen concentration, temperature, and photosynthetic CO_2_ uptake. These factors contributed differently to enzymatic activity within the biofilm matrix and, consequently, to variations in biofilm mass and structure.

### 3.4. Corrosion Tests: Measurement of Volumetric Index, Hydrogen Release Volume, and Penetration Index (Ip)

In general, an inhibitor, such as a toxic substance, can inhibit a microorganism’s cellular processes, decreasing its metabolic activity. Thus, cellular multiplication slows, but cells can quickly adapt to chemical stress and develop resistance to the inhibitory substance. Inhibition of metal corrosion with the help of microorganisms is useful because microorganisms form a passivating film on the metal surface, protecting it against the corrosive action of ions dissolved in the reaction medium [46].

**Figure 10:**
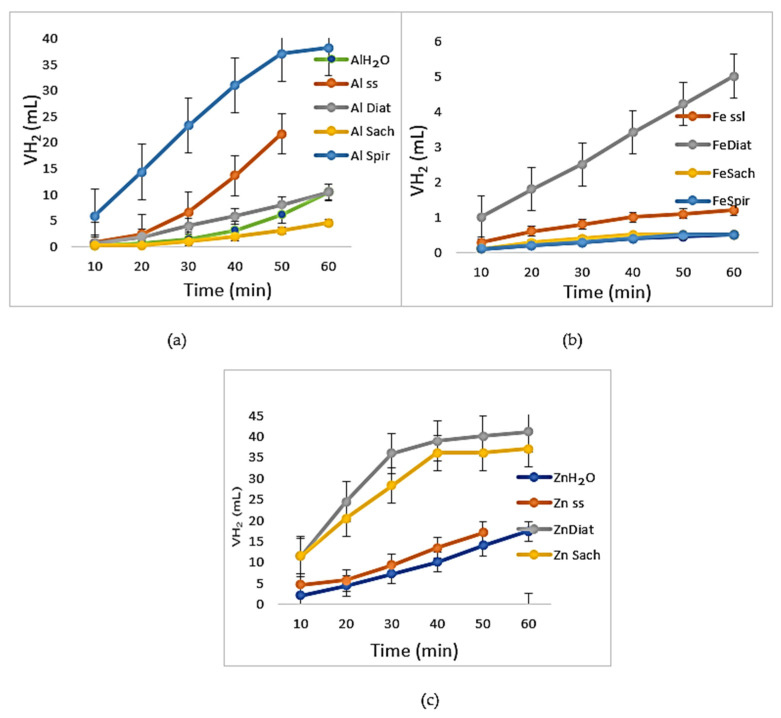
Hydrogen evolution volume during the reaction of tested coupons with HCl in the presence of Al (**a**), Fe (**b**), and Zn (**c**) (measured values are presented with standard deviation (*n* = 4)).

Aluminum is a metal whose corrosion resistance largely depends on the stability of its protective oxide layer (Al_2_O_3_). In the case of the Al–*Spirulina* system (Al–*_Spir_*), a very steep slope was observed in the hydrogen evolution curve, reaching approximately 38 mL H_2_. This behavior suggests that prior immersion in the *Spirulina* suspension either disrupted the protective oxide layer or generated a porous and heterogeneous surface that facilitated subsequent acid attack. Under these conditions, *Spirulina* appeared to accelerate aluminum corrosion, acting as an aggressive depassivating agent. In comparison, the aluminum sample immersed in saline solution (Al*_SS_*) exhibited moderate corrosion, with hydrogen evolution of approximately 22 mL H_2_. The presence of chloride ions is known to have a localized destructive effect on the aluminum oxide film, promoting pitting corrosion (Figure 10). Overall, these results indicate that both biological activity and chloride-containing environments can compromise the passive layer of aluminum, enhancing corrosion processes through different mechanisms [39].

The Al*_H_*_2_*_O_*, Al*_Sach_*, and Al*_Diat_* (Figure 10a) samples exhibited comparatively low corrosion rates. The *Saccharomyces* and *Diatom* media showed behavior similar to that of distilled water, indicating that pre-treatment with these biological suspensions maintained corrosion kinetics at minimal levels. In these cases, the oxide layer on aluminum appeared to remain relatively stable [41,42,43]. The presence of salts (Al*_SS_*) promoted the formation or stabilization of a surface layer that partially inhibited active anodic sites and reduced the cathodic reaction rate in acidic conditions. However, although the Al*_ss_* curve displayed approximately linear kinetics, the corrosion rate remained higher than in the control (Al*_H_*_2_*_O_*), suggesting that chloride ions still contributed to localized depassivation. As shown in Figure 10b, iron exhibited significantly higher resistance to acidic attack compared to aluminum and zinc. In the Fe*_Diat_* system, the *diatom* medium activated the iron surface, creating conditions that facilitated acid attack, resulting in a hydrogen evolution volume of approximately 5 mL. For Fe*_ss_*, the saline medium slightly accelerated corrosion compared to distilled water, but the effect was much less pronounced than in the presence of *Diatoms* [41]. In contrast, *Spirulina* and *Saccharomyces* had a neutral or slightly protective effect on iron, similar to distilled water, with hydrogen evolution remaining below 1 mL, indicating negligible corrosion.

Figure 10c shows that zinc was highly reactive in acidic media, in contrast to aluminum. In the Zn*_Diat_* and Zn*_Sach_* systems, these biological media significantly accelerated corrosion, increasing hydrogen evolution to approximately 40 mL. Zinc exposed to *Diatoms* and *Saccharomyces* suspensions exhibited the steepest initial slopes, reflecting a rapid corrosion rate. Notably, the saline medium had a less aggressive effect on zinc compared to the biological media [44,46].

Overall, the results highlight distinct metal–microorganism interactions, with biological media exerting either protective or activating effects depending on the specific metal and environmental conditions.

**Figure 11:**
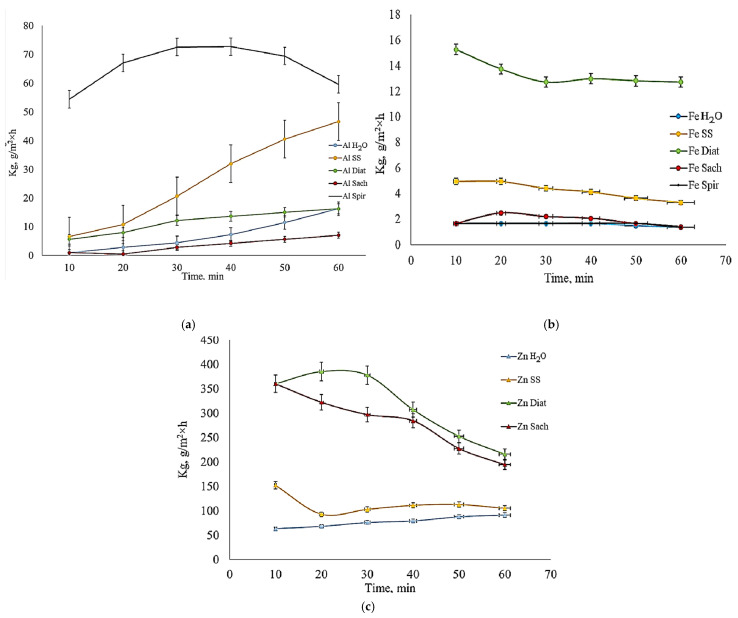
Gravimetric index (Kg) of Al (**a**), Fe (**b**), and Zn (**c**) treated samples measured over time in 3%HCl (measured values are presented with standard deviation (*n* = 4)).

*Diatoms* (Diat) and *Saccharomyces* (Sach) cultures formed a protective film on the aluminum surface (Figure 11a), acting as a barrier that limited direct contact between the metal and the HCl solution. In contrast, *Spirulina* (Spir) intensified aluminum corrosion during the initial stage of contact with HCl. Subsequently, the time-dependent corrosion curve reached a plateau, and toward the end of the testing interval, the corrosion rate decreased. This reduction may be attributed to the formation of a secondary protective layer on the aluminum surface. The presence of a *Diatoms* biofilm significantly reduced aluminum degradation. The slight upward trend observed in the time-dependent corrosion rate curve can be explained by the gradual deterioration of the protective film over time.

In the case of iron (Figure 11b), *Spirulina* demonstrated a protective effect. The corrosion rate remained approximately constant, with a slight decrease after 60 min. This behavior may be attributed to the inhibitory effect of *Spirulina* in acidic environments, as reported in the literature [51,61,62,63]. In contrast, the Fe*_Diat_* sample exhibited the highest corrosion rate in acidic conditions, indicating that *Diatom* biofilm acted as a corrosion accelerator. Although the time-variation curve showed a slight downward trend, the corrosion rate remained relatively high. This may be explained by the ability of the biofilm formed on the iron surface to retain aggressive species from the environment, thereby sustaining localized corrosion.

The corrosion rates of samples treated with *Spirulina* and *Saccharomyces* were similar to those observed in distilled water. However, the increase in Cl^−^ concentration in the corrosive medium caused the Fe*_ss_* sample to exhibit a higher corrosion rate than most biological treatments, with the exception of *Diatoms*. The general downward trend in corrosion rate over time can also be attributed to the depletion of oxidizing agents and active ionic species in solution.

For zinc (Figure 11c), *Spirulina* acted as a strong corrosion accelerator, leading to complete consumption of the specimen within the first 10 min of immersion in HCl. For this reason, the Zn*_Spir_* sample does not appear in the diagram. *Diatoms* and *Saccharomyces* cultures also accelerated zinc corrosion. Upon contact with HCl, the initially formed protective layer was rapidly destroyed, resulting in corrosion rates on the order of hundreds of g/m^2^·h. Although the corrosion rates remained within the same order of magnitude, the time-dependent curves exhibited a downward trend, suggesting partial restoration of a protective layer at a rate slower than the ongoing corrosion process. Overall, the biological media exhibited significantly higher corrosive potential than saline solution or distilled water. Analysis of the time evolution of the penetration index for aluminum showed that *Spirulina* generated the most aggressive acidic environment. The time-variation curve of the penetration index for the *Spir*-treated sample followed a quadratic polynomial model, whereas for all other treatment environments, the penetration index varied linearly with time.

The penetration indices for Zn*_Sach_* and Zn*_H_*_2_*_O_* exhibit linear time dependence. In contrast, Zn*_Diat_* and Zn*_ss_* display quadratic and cubic polynomial trends, respectively. For Fe, the penetration indices are best represented by second- and third-order polynomials, except for *SS*, which shows a linear relationship. In the case of *Spirulina*, linearity is interrupted solely by the penetration index observed at 60 min of immersion in HCl.

**Figure 12:**
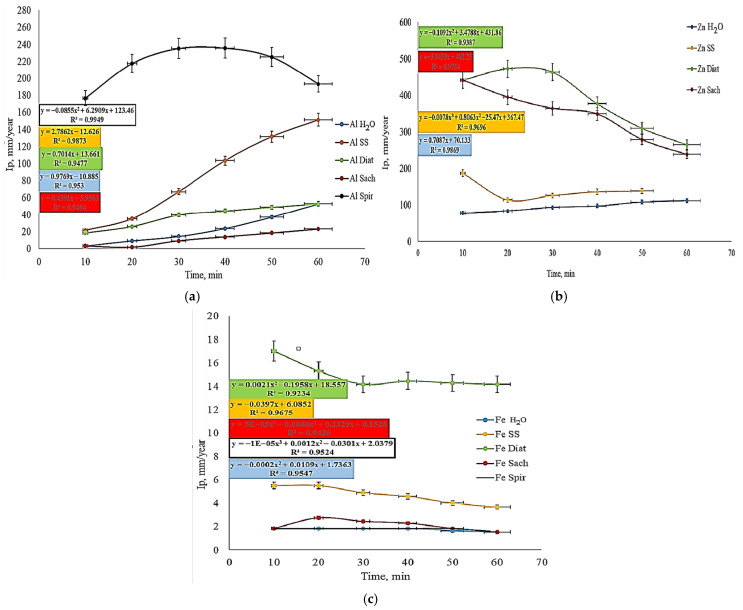
Corrosion rate evolution (I_p_) of (**a**) Al, (**b**) Zn and (**c**) Fe in 3% w HCl after immersion in various initial media (measured values are presented with standard deviation (*n* = 4)).

**Figure 13:**
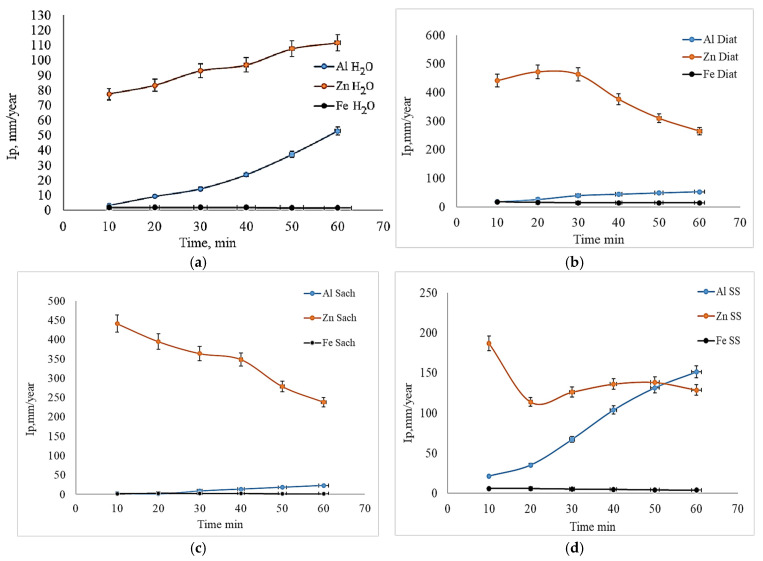
Variation in Ip for plates used as blank samples (**a**), metallic plates developed in *Diatoms* suspension (**b**), metallic plates developed in Saccharomyces suspension (**c**), and metallic plates developed in saline solution (**d**) (measured values are presented with standard deviation (*n* = 4)).

Upon analysis of the corrosion index (Ip) for aluminum, zinc, and iron plates in HCl, after initial exposure in different environments, Figure 12 and Figure 13 show that corrosion resistance depends on both the nature of the metal and the composition of the environment. Iron proved to be the most resistant, presenting a very low Ip in all environments, even in salt water. Aluminum was sensitive to certain biological environments, and the highest corrosion was recorded in *Spirulina*, demonstrating that not all biological environments protect aluminum [58,61]. *Diatoms* and *Saccharomyces* suspensions had an inhibitory effect. Zinc is the most vulnerable metal, with maximum corrosion in *Spirulina*, followed by salt water, and biological environments partially reduce the corrosion rate. These observations align with recent trends regarding metal protection and the use of ecological inhibitors.

From the chemical analysis values of the tested reactors, we observe that oxygen variation is influenced by pH changes in the reaction environment. The ability of the reaction medium to form films on the metal surface depends on the content of dissolved chemical species and the reaction conditions (stirring time, reaction temperature). The literature indicates that zinc stored in water with high mineral content has a corrosion rate of 15 µm/year, compared to 150 µm/year in water without minerals [45]. The chemical reactions that intervene in this process are important because, together with the chemical environment in which the exposed plates are found, they can form protective surfaces against corrosion. The oxygen content of the reaction medium also contributes to increasing the corrosion rate. The presence of oxygen in the reaction medium depolarizes and accelerates the chemical reactions by combining with the hydrogen formed, as shown in the following reactions [41,45] (Equations (15)–(20)):

(15) $$4\mathrm{Fe}+3O_{2}+6H_{2}O\to 4\mathrm{Fe}{\left(\mathrm{OH}\right)}_{3}↓$$

(16) $$3\mathrm{Fe}+4H_{2}O\to {\mathrm{Fe}}_{3}O_{4}+4H_{2}↑$$

(17) $$3\mathrm{Fe}+2O_{2}\to {\mathrm{Fe}}_{3}O_{4}$$

(18) $$Zn+2H_{2}O\to Zn{\left(OH\right)}_{2}+H_{2}↑$$

(19) $$H_{2}+\frac{1}{2}O_{2}\to H_{2}O$$

(20) $$4\mathrm{Al}+3O_{2}\to 2{\mathrm{Al}}_{2}O_{3}$$

Oxygen was removed from the reaction medium using algal suspensions, which consumed oxygen via cellular processes that released CO_2_. The inhibition of corrosive agents on metals will be examined using both electrochemical and radiochemical techniques. The resulting reaction product may form chemical bonds in which carbon monoxide functions as an electron donor and the metal acts as an electron acceptor, thereby potentially inhibiting corrosion [60]. This process is attributed to the ability of certain metals, such as zinc, to form stable carbonyl complexes.

The *Spirulina*-containing medium exhibited the most aggressive behavior toward aluminum, selectively disrupting its passive oxide layer, while exerting a protective effect on iron. In contrast, *Diatom*-containing media caused severe corrosion of zinc and were the only environments to produce visible attack on iron, whereas aluminum remained largely unaffected. This behavior suggests a combined mechanical–chemical mechanism, linked to the siliceous structure of *Diatoms* and the formation of differentiated aeration cells. The *Saccharomyces*-containing medium exerted a dual effect, acting as a corrosion inhibitor of aluminum and iron while significantly accelerating zinc corrosion, most likely through chelation and metabolic acidification. Hydrogen evolution analysis confirmed a strict dependence on the metallic substrate, with iron exhibiting hydrogen volumes an order of magnitude lower than those of zinc and aluminum, reflecting its superior thermodynamic stability under the tested conditions [19,61]. For zinc, biological media promoted generalized anodic dissolution, whereas chloride ions from saline pre-treatment induced localized pitting corrosion, structurally more critical despite being associated with lower overall hydrogen evolution. In the case of aluminum, polarization curves revealed rapid passive-layer breakdown in the *Spirulina* medium, followed by controlled diffusion-limited behavior, whereas significant iron susceptibility was observed exclusively in the *Diatoms* medium [62,63,64].

### 3.5. Microscopic View: Optical and Scanning Electron Microscopy Examinations

**Figure 14:**
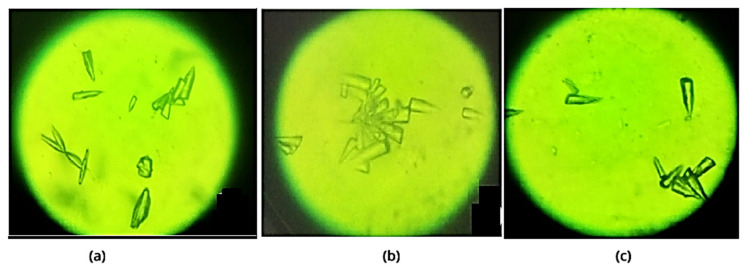
Microscopic observations on the evolution of the *Diatoms* suspension after 28 days of contact with the metal plates. Optical light microscope imagery at 250× magnification of *Diatoms* in the environment created by the presence of the Al (**a**), Fe (**b**), and Zn (**c**) plates.

**Figure 15:**
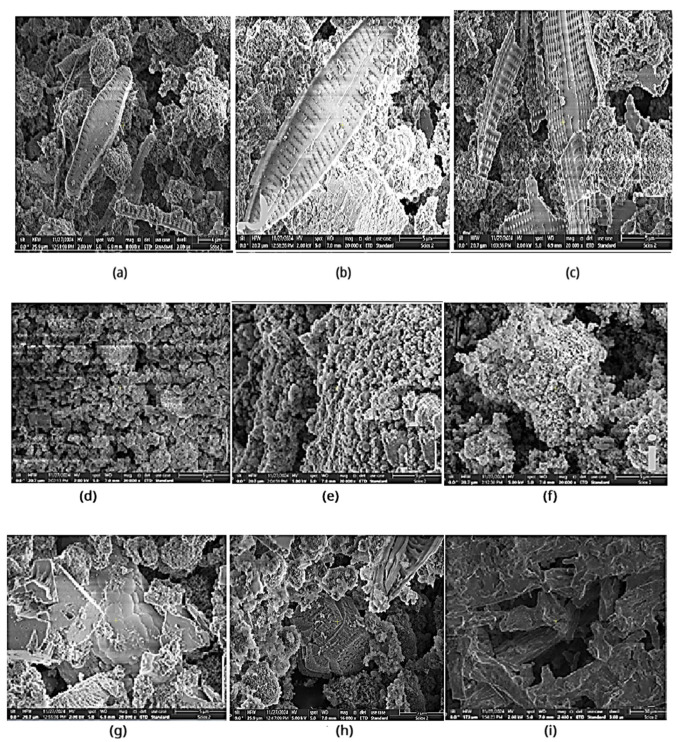
Scanning electron microscope imagery of the DSS in the presence of different Al, Zn, and Fe plates. Surface structure in culture grows after 28 days of contact: *Diatoms* (**a**–**c**), *Spirulina* (**d**–**f**), *Saccharomyces* (**g**–**i**).

Analysis of samples demonstrated that *diatom* algae exhibited increased cell growth in the presence of Zn plates compared to media containing Al or Fe plates (Figure 14). The cell wall and internal structures remained intact under all tested conditions (Figure 15). These findings indicate that metal ions do not adversely affect cell growth or cell wall integrity, suggesting that *diatom* algae possess mechanisms to resist and adapt to chemical stress, potentially through cellular protection processes. The silica content of their cell walls may contribute to this resistance, while the algae continue to convert dissolved carbon dioxide into oxygen via photosynthesis [13,32,33,34,35,36,37,38].

## 4. Conclusions

This study investigates how microorganisms inhibit corrosion of aluminum, iron, and zinc by forming passivating films on their surfaces. Results indicate that *diatom* algae multiply most rapidly on aluminum, followed by iron and zinc.

The metal type influenced cellular viability of the studied microorganisms. During the test, in similar conditions, microorganisms form different amounts of biofilm.

*Spirulina* exhibited the strongest aggressive effect on aluminum by selectively disrupting its passive oxide layer. In contrast, *Spirulina* protected iron under the same conditions, highlighting distinct interactions with each metal’s surface chemistry.

Media containing *diatoms* caused severe corrosion of zinc and were the only conditions that visibly degraded iron, while aluminum remained unaffected. This effect may be due to the siliceous structure of *diatoms*, which can promote differential aeration cells and localized corrosion.

The medium containing *Saccharomyces* had a dual effect: it inhibited corrosion on aluminum and iron but accelerated zinc corrosion, likely due to chelation and metabolic acidification. The impact of the environment on metal corrosion depends on the specific metal–environment combination. Results show that saline environments accelerate corrosion of reactive metals, while biological environments can be either protective or aggressive, depending on the metal–microorganism interaction. *Spirulina* may promote acid-mediated corrosion of aluminum, increasing corrosion rates that can sometimes exceed those caused by *Saccharomyces*.

## Figures and Tables

**Table 1:** Presentation of the bioreactor system for 28-day experimental evaluation.

Bioreactors					Metal Plate	Series
1 Al*_H_*_2_*_O_*	4 Al*_ss_*	7 Al*_Diat_*	10 Al*_Sach_*	13 Al*_Spir_*	Al	A
2 Zn*_H_*_2_*_O_*	5 Zn*_ss_*	8 Zn*_Diat_*	11 Zn*_Sach_*	14 Zn*_Spir_*	Zn	B
3 Fe*_H_*_2_*_O_*	6 Fe*_ss_*	9 Fe*_Diat_*	12 Fe*_Sach_*	15 Fe*_Spir_*	Fe	C
**Reaction Environment**						
H_2_O	NaCl 3%	*Diatoms*	*Saccharomyces c.*	*Spirulina p.*		

## Data Availability

The original contributions presented in this study are included in the article. Further inquiries can be directed to the corresponding authors.

## Abbreviations

The following abbreviations are used in this manuscript:

DO	Dissolved oxygen
DSS	*Diatoms, Saccharomyces, Spirulina*
Ip	Penetration index
Ic	Corrosion index
Kg	Gravimetric index
OD600	Optical density
QS	Quorum sensing
MIC	Microbiologically influenced corrosion
MM	Mineral medium
OECD	Guidelines for the testing of chemicals of the Organization for Economic Cooperation and Development
